# Phytochemical Screening and Antinociceptive and Antidiarrheal Activities of Hydromethanol and Petroleum Benzene Extract of* Microcos paniculata* Barks

**DOI:** 10.1155/2016/3167085

**Published:** 2016-09-29

**Authors:** Rafath Ara Moushome, Mst. Irin Akter, Md. Abdullah Aziz

**Affiliations:** ^1^Department of Pharmacy, Stamford University Bangladesh, 51 Siddeswari Road, Dhaka 1217, Bangladesh; ^2^Department of Pharmacy, Jessore University of Science & Technology, Jessore, Bangladesh

## Abstract

*Introduction*.* Microcos paniculata* is traditionally used for treating diarrhea, wounds, cold, fever, hepatitis, dyspepsia, and heat stroke.* Objective*. To investigate the qualitative phytochemical constituents of hydromethanol (HMPB) and petroleum benzene extract of* Microcos paniculata* barks (PBMPB) and to evaluate their antinociceptive and antidiarrheal activities.* Methods*. Phytochemical constituents and antinociceptive and antidiarrheal activities were determined and evaluated by different tests such as Molisch's, Fehling's, Mayer's, Wagner's, Dragendorff's, frothing, FeCl_3_, alkali, Pew's, and Salkowski's test, general test of glycosides, Baljet and NH_4_OH test, formalin-induced paw licking, acetic acid-induced writhing, tail immersion, and hot plate tests, and castor oil and MgSO_4_ induced diarrheal tests.* Results*. These extracts revealed the presence of saponins, flavonoids, and triterpenoids and significantly (^⁎^ *P* < 0.05, versus control) reduced paw licking and abdominal writhing of mice. At 30 min after their administration, PBMPB revealed significant increase in latency (^⁎^ *P* < 0.05, versus control) in tail immersion test. In hot plate test, HMPB and PBMPB 200 mg/kg showed significant increase in response latency (^⁎^ *P* < 0.05, versus control) at 30 min after their administration. Moreover, both extracts significantly (^⁎^ *P* < 0.05, versus control) inhibited percentage of diarrhea in antidiarrheal models.* Conclusion.* Study results indicate that* M*.* paniculata* may provide a source of plant compounds with antinociceptive and antidiarrheal activities.

## 1. Introduction

Plants provide complicated, mixed, and distinct nonnutrient elements which act as the main basis of drug discovery [1]. Plant extracts contain phytochemical constituents for miscellaneous medicinal activities which are bioactive in nature [2].

Very unpleasant emotional and sensory events accompanied by definite or probable tissue damage characterize nociception [3]. Tissue damage may occur due to different reasons such as thermal, chemical, and mechanical incitements or the existence of pathologic procedure- inflammation, tumor, nerve damage, and muscle spasm. Pain can be managed by steroidal and nonsteroidal analgesics. Being oldest analgesics, the steroidal form (opioids) can lessen cancer and postoperative associated pains, acute and chronic pains which are deep and serious in nature. Though beneficial, various adverse effects such as gastric lesions as well as tolerance and dependence are experienced by steroidal and nonsteroidal anti-inflammatory drugs (NSAIDs) [4]. Therefore, it is extremely needed to discover novel antinociceptive agents with similar or higher activity than presently used drugs but with less toxic effects.

Mainly children below 5 years of age suffer from malnutrition caused by diarrhea. They experience tremendous mortality and morbidity also. Research revealed that several microorganisms like* Salmonella, Escherichia coli, Vibrio cholerae, and Shigella* generate and discharge enterotoxins which are the major cause of diarrhea in developing countries [5]. Antidiarrheal agents can be obtained by using plants as a key source [6].

Kathgua or Fattashi is the local name of* Microcos paniculata* (family: Tiliaceae) in Bangladesh that are harvested all over Bangladesh. Generally it develops naturally as a shrub or short tree. This plant is known for many traditional uses, for example, to treat diarrhea, wounds, cold, fever, hepatitis, dyspepsia, and heat stroke. Moreover it has insecticidal activity. However, it is active against the digestive system also. Thorough study of literature revealed that it showed several activities, including analgesic, antimicrobial, neuropharmacological, *α*-glucosidase inhibition, brine shrimp lethality, free radical scavenging, antipyretic, nicotinic receptor antagonistic, larvicidal, cytotoxic, insecticidal, anti-inflammatory, and antidiarrheal activities. In addition, it can prevent angina pectoris, coronary heart disease, or coronary artery disease or ischaemic heart disease. Acute toxicity study was also carried out [2, 7–11].

So, the present study was designed to identify phytoconstituents and to evaluate the antinociceptive and antidiarrheal activities of hydromethanol (HMPB) and petroleum benzene extract of* Microcos paniculata* barks (PBMPB).

## 2. Materials and Methods

### 2.1. Chemicals and Reagents

Aspirin, diclofenac sodium, tramadol hydrochloride, and paracetamol were purchased from the Bangladeshi manufacturer Gonoshasthaya Pharmaceuticals Ltd., Square Pharmaceuticals Ltd., Acme Laboratories Ltd., and Beximco Pharmaceuticals Ltd., respectively. All solvents used were of analytical grade and obtained from Merck, Germany.

### 2.2. Plant Material

*M. paniculata* barks were collected from the Jahangirnagar University Campus (23.8791°N, 90.2690°E), Savar, Dhaka, Bangladesh, in November 2013. Species identification was verified by Sarder Nasir Uddin, Principal Scientific Officer at the Bangladesh National Herbarium (accession number 35348). A dried specimen was deposited in the herbarium for future reference.

### 2.3. Preparation and Extraction of Plant Material

Hydromethanolic (mixture of 80% methanol and 20% water) and petroleum benzene extractions were performed separately on 275 g and 150 g of powdered barks of* M. paniculata*. Fresh barks were rinsed 3-4 times successively with running water and once with sterile distilled water. Washed plant materials were then dried in the shade for a period of 7 d. The dried plant materials were then ground by using a laboratory grinding mill (MACSALAB 200 Cross Beater, Eriez, Erie, Pennsylvania, USA) and passed through a 40-mesh sieve to get fine powder. Powdered barks (275 g and 150 g) were separately dissolved in hydromethanol (2200 mL methanol and 550 mL water) and petroleum benzene (1500 mL) in closed containers and occasionally stirred for 15 d. Then extractions were completed by using rotary evaporator (RE601, Yamato Scientific America Inc., Santa Clara, California, USA) at a temperature of 40°C. Sterile cottons followed by Whatman No. 1 filter papers were used to filter the liquid extracts. The filtrates were then dried in a hot air oven (BST/HAO-1127, Bionics Scientific Technologies Pvt. Ltd., Delhi, India) at 40°C. The extraction yields of HMPB and PBMPB were 10.30% (w/w) and 1.39% (w/w), respectively. Both extracts were stored at 4°C for additional studies.

### 2.4. Phytochemical Screening

Freshly prepared HMPB and PBMPB were subjected to different qualitative tests according to Aziz and Billmary et al. [2, 12] to find out the presence of phytoconstituents like carbohydrates, alkaloids, saponins, tannins, flavonoids, triterpenoids, glycosides, and anthraquinones, through characteristic color changes.

### 2.5. Experimental Animals

One hundred and ninety-five* Swiss albino* mice of either sex, 6-7 weeks, weighing 25–30 g were collected from the Department of Pharmacy, Jahangirnagar University, Savar, Dhaka, Bangladesh, which were used in these experiments. These animals were kept under standard environmental conditions, having relative humidity 55%–65%, 12 h light/12 h dark cycle, and (27.0 ± 1.0)°C temperature. Proper supplies of foods and water* ad libitum* were ensured. Before the experiment, animals were adapted to the laboratory conditions for 1 week. The Institutional Animal Ethical Committee of Stamford University Bangladesh approved the protocol (protocol no. SUB/IAEC/1765) used in the experiments conducted with these animals.

### 2.6. Acute Toxicity Study

Generally acute toxicity results within a short time (normally less than 24 h) and is regarded as the expression of adverse effects because of a single exposure or multiple exposures of a substance. It follows the guidelines of Organization of Economic Cooperation and Development (OECD) for determining the half lethal dose (LD_50_) of the experimental samples [2, 13]. In total, fifteen mice were separated into three groups: control group and test groups (HMPB and PBMPB), having five animals per group. The experimental samples (HMPB and PBMPB) were administered orally at different concentrations (100, 250, 500, 1000, 2000, 3000, and 4000 mg/kg body weight). Then some parameters were checked such as mortality, diarrhea, noisy breathing, salivation, convulsion, injury, changes in locomotor activity, weakness, discharge from eyes and ears, coma, pain, aggressiveness, food or water refusal, or any signs of toxicity in each group of animals after observing them every 1 h for next 5-6 h. Moreover, each group of animals were noticed for 2 weeks for concluding assessment [2, 13].

### 2.7. Antinociceptive Study

#### 2.7.1. Formalin-Induced Paw Licking Test

The method of Hunskaar and Hole [14] was used for the paw licking study. Thirty mice were divided into control group (distilled water), positive control or standard group (diclofenac sodium (DS), 100 mg/kg body weight), and test groups (HMPB and PBMPB at 200 and 400 mg/kg body weight), containing five mice in each group. The animals were fasted for 16 h with water* ad libitum*. Mice in the control group, positive control group, and test groups received one dose of distilled water, diclofenac sodium, HMPB, and PBMPB orally. After 1 h of treatment of each group, 2.7% formalin (v/v) at a dose of 20 *μ*L was injected into the dorsal surface of the left hind paw of each mouse. The time spent for licking the injected paw was recorded. Animals were observed for 5 min after formalin injection (acute phase) and for 5 min in delayed phase, which was starting at the 20th minute after formalin injection. The percentage of inhibition of licking was calculated using the following formula:

(1) Inhibition %=1−Licking  time  (extract  or  standard  drug)Licking  Time  (normal  control)×100.

#### 2.7.2. Acetic Acid-Induced Writhing Test

The method of Koster et al. [15] was employed for the writhing test. The animals were fasted for 16 h with water* ad libitum*. Mice were pretreated with extracts as mentioned before. DS (100 mg/kg) was used as standard or positive control and distilled water as normal control. Forty-five minutes later, each mouse was injected intraperitoneally with 0.7% acetic acid at a dose of 10 mL/kg body weight. Fifteen minutes after the administration of acetic acid, the number of writhing responses was recorded for each animal during a 5-minute period. The percentage of inhibition of writhing was calculated using the following formula:

(2) Inhibition %=1−No.  of  writhing  (extract  or  standard  drug)No.  of  writhing  (normal  control)×100.

#### 2.7.3. Tail Immersion Test

The method of Toma et al. [16] was employed for this test. The method was used to evaluate the central mechanism of analgesic activity. Here the painful reactions in animals were generated by thermal stimulus through dipping the tip of the tail in hot water. Mice were grouped and treated as described before. Tramadol (10 mg/kg) was used as the reference drug. The animals were fasted for 16 h with water* ad libitum*. Before and after the treatment of each group, the basal reaction time, that is, time taken (in second) to withdraw it from hot water source, was measured by immersing the tail tips of the mice (last 1-2 cm) in hot water of (55 ± 1)°C and the results were compared with control group. A latency period of 15 s was set as the cutoff point to avoid injury to mice. The latent period of the tail-flick response was determined before 30 min and after 30, 60, 120, and 180 min of the respective treatment of each group.

#### 2.7.4. Hot Plate Test

Hot plate test was performed according to the method of Turner [17]. The method was used to evaluate the central mechanism of analgesic activity [18]. At first, mice were screened for this test by inserting them on a hot plate individually that was kept at (55 ± 1)°C. The mice showing initial reaction time (difference of time between the placement of mice on hot plate and their responses to occur) of 15 s or less were selected for this study. A cutoff point of 15 s was used to avoid the damage to the paw. Mice were grouped and treated as described before. Tramadol (10 mg/kg) was used as the reference drug. The animals were fasted for 16 h with water* ad libitum*. 30 min before the treatment of each group, the response latencies of mice were measured by placing them on hot plate after the observations of some parameters such as removal, jumping, or licking of the paws. The response latencies were also recorded after 30, 60, 120, and 180 min of the respective treatment of each group.

### 2.8. Antidiarrheal Study

#### 2.8.1. Castor Oil Induced Antidiarrheal Test

The model of Shoba and Thomas was followed for carrying out the test [19]. Preliminary screening of animals was done by administering 0.5 mL of castor oil orally and those animals that started diarrhea were selected finally for the test. Thirty mice were divided into control group (distilled water), positive control or standard group (Loperamide HCl, 3 mg/kg body weight), and test groups (HMPB and PBMPB at 200 and 400 mg/kg body weight), containing five mice in each group. The animals were fasted for 16 h with water* ad libitum*. Mice in the control group, positive control group, and test groups received one dose of distilled water, Loperamide HCl, HMPB, and PBMPB orally. After 30 min of the above treatments, each animal received 0.5 mL of castor oil orally for initiating diarrhea. Blotting paper lined individual cage was used for placing every animal. These blotting papers were changed at every hour. The number of diarrheal feces was recorded for a period of 4 h and the percentage of inhibition of defecation was calculated for every group of animals.

#### 2.8.2. MgSO_4_ Induced Antidiarrheal Test

A similar procedure as for castor oil induced diarrhea was maintained for magnesium sulphate induced diarrheal model. Preliminary screening of animals for diarrhea was done by administering magnesium sulphate at a dose of 2 g/kg orally. Then, the animals were fasted for 16 h with water* ad libitum*. Mice were grouped and treated as described before. 30 min later of pretreatments, magnesium sulphate was administered orally at a dose of 2 g/kg to the animals and the antidiarrheal activity was expressed by comparing the percent of inhibition of defecation of different groups with control group [20].

### 2.9. Statistical Analysis

All results are expressed as mean ± standard error (SE). All tests were analyzed statistically by one-way ANOVA followed by Dunnett's *t*-test. In addition, the results of tail immersion and hot plate test were analyzed by using repeated measure ANOVA (RM-ANOVA). In case of all in vivo studies, pairwise comparison of means among the groups (except control) was done by one-way ANOVA followed by post hoc Tukey's HSD test. *P* < 0.05 was considered to be statistically significant. All data were analyzed using SPSS software (version 16; IBM Corporation, New York, USA).

## 3. Results

### 3.1. Phytochemical Screening

It is important to depict the chemical nature of plant materials, when their pharmacological activities are evaluated [2]. Phytochemical screening of the HMPB and PBMPB showed the presence of very few secondary metabolites or phytoconstituents, which are summarized in Table 1. There was no primary metabolite in HMPB and PBMPB. In addition, HMPB and PBMPB were shown to have different compositions. Both HMPB and PBMPB showed the presence of saponins and triterpenoids. However, flavonoids test did not show consistent results. Flavonoids content in HMPB was indicated by Pew's test, but not by alkali test. Moreover, alkaloids, tannins, glycosides, and anthraquinones were absent in both HMPB and PBMPB.

### 3.2. Acute Toxicity Study

No mortality or signs of toxicity or behavioral changes were observed during the 14-day observation period in mice receiving doses up to 4000 mg/kg of HMPB or PBMPB (test groups). The control group showed the same result. This demonstrates that the test groups did not experience acute oral toxicity at the doses tested.

### 3.3. Antinociceptive Study

#### 3.3.1. Formalin-Induced Paw Licking in Mice

Of the plant extracts, PBMPB at 400 mg/kg body weight showed significant highest percentage of inhibition (86.56 ± 0.27%) of paw licking in mice during the late phase of formalin injection (^*∗*^ *P* < 0.05, versus control). In addition, DS was effective at both acute and delayed phase. Again, PBMPB at 400 mg/kg revealed a little increase of percentage of inhibition of paw licking from acute phase to delayed phase. But, percentage of inhibition of paw licking was decreased in late phase for HMPB and PBMPB at 200 mg/kg, respectively (Table 2).

#### 3.3.2. Acetic Acid-Induced Writhing Test

In the mouse writhing assay, all groups caused significant percentage inhibition of writhing (^*∗*^ *P* < 0.05, versus control). Treatment with DS (100 mg/kg) resulted in less writhing than treatment with either of the extracts and either of the doses. The maximum percentage of inhibition of writhing resulting from treatment with plant extracts (73.98 ± 1.24%) was obtained by HMPB at 400 mg/kg. Standard drug, DS, at 100 mg/kg body weight showed (87.68 ± 1.36%) writhing inhibition (Table 3).

#### 3.3.3. Tail Immersion Test

At 30 min after their administration, the tramadol group and PBMPB had significant increase in latency (^*∗*^ *P* < 0.05, versus control). The maximum effects of the extracts, without HMPB 400 mg/kg, were obtained at 30 min (^*∗*^ *P* < 0.05, versus control). But HMPB did not demonstrate any significant increase in latency at any time point (^*∗*^ *P* < 0.05, versus control). It was observed that tramadol and PBMPB 400 mg/kg showed significant latency at 30, 60, 120, and 180 min, respectively (^*∗*^ *P* < 0.05, versus control at each of the cases). In addition, PBMPB 200 and 400 mg/kg were active at late phase (at 120 min) of tail immersion test (Table 4).

#### 3.3.4. Hot Plate Test

After the administration of tramadol group, HMPB, and PBMPB 200 mg/kg, they showed significant increase in response latency (^*∗*^ *P* < 0.05, versus control) at 30 min. The maximum effects of the extracts, without PBMPB 400 mg/kg, were obtained at 30 min (^*∗*^ *P* < 0.05, versus control). It was observed that all of the extracts and tramadol showed significant response latency at 30, 60, 120, and 180 min, respectively (^*∗*^ *P* < 0.05, versus control at each of the cases). In addition, HMPB 200 mg/kg and PBMPB 200 mg/kg were active at late phase (at 120 and 180 min) of hot plate test. However, HMPB 400 mg/kg showed late phase activity at 180 min (Table 5).

### 3.4. Antidiarrheal Test

#### 3.4.1. Castor Oil Induced Diarrheal Test

In case of castor oil induced diarrheal test, Loperamide HCl, HMPB, and PBMPB 200 and 400 mg/kg produced antidiarrheal effect in mice. All of the extracts at doses of 200 and 400 mg/kg significantly decreased (^*∗*^ *P* < 0.05, versus control) the total number of diarrheal feces. Highest and significant (^*∗*^ *P* < 0.05, versus control) percentage of inhibition of diarrhea (62.95 ± 3.23%) was revealed by HMPB 400 mg/kg (Table 6).

#### 3.4.2. MgSO_4_ Induced Diarrheal Test

In case of MgSO_4_ induced diarrheal test, Loperamide HCl, HMPB, and PBMPB produced antidiarrheal effect in mice. All of the extracts at doses of 200 and 400 mg/kg significantly decreased (^*∗*^ *P* < 0.05, versus control) the total number of diarrheal feces. Highest and significant (^*∗*^ *P* < 0.05, versus control) percentage of inhibition of diarrhea (68.13 ± 6.13%) was revealed by PBMPB 400 mg/kg (Table 7).

## 4. Discussion

Plants may face biotic stress (living organisms, such as bacteria, fungi, parasites, viruses, harmful and beneficial insects, native or cultivated plants, and weeds creating biotic stress on plants by injuring them) or abiotic stress (happening on plants and animals triggering harm to them by nonliving factors such as salinity, drought, sunlight, wind, deficiency of nutrients in soil, and overwatering) [21–23]. Because of these stresses, they synthesize phytochemicals which have health stimulating effect. As phytochemicals have health stimulating effect it is vital to increase their intake in our diet [23]. Besides, initial screening of secondary metabolites assists the detection of bioactive compounds which initiates drug discovery and development [2].

Many people intake medicinal plants. However, severe toxicities can arise by using some of these plants. So, their toxicological studies must be performed [24]. Moreover, toxicity data of various plants are not available [25]. Through acute toxicity study, primary data on the toxic effect of any compound can be obtained after the single administration of that compound [26]. However, suitable range of doses of the materials for successive usage can be obtained by acute oral toxicity studies [2]. LD_50_ of the plant extracts could not be obtained, as no mortality was observed up to the dose as high as 4000 mg/kg and the extracts were found to be safe with a broad therapeutic range. Therefore, two comparatively high doses (200 and 400 mg/kg) for both HMPB and PBMPB were used for in vivo doses.

Formalin-induced paw licking test is involved in the determination of central as well as peripheral activities of nociception. This licking test involves two dissimilar licking phases. One is known as early phase (0–5 min after the injection of formalin) and the other is late phase (20–30 min after the injection of formalin). In case of early phase, nociceptors are straightly affected by formalin and this phase is also called as noninflammatory or neurogenic pain, whereas inflammatory pain occurs from late phase of formalin-induced pain. This neurogenic pain starts through the participation of substance *P*, but inflammatory pain starts with the release of prostaglandins (PGs), serotonin, bradykinin, and histamine. Centrally acting drugs as opioids can inhibit the pain of both early and late phase by acting on the CNS. Moreover, the narcotic analgesics contribute to the inhibition of early and late phase pain. Peripheral analgesics as acetylsalicylic acid can inhibit the pain of late phase [2, 27]. The abilities of HMPB and PBMPB to inhibit both phases of formalin-induced paw licking suggest its central and peripheral activities as well as its capacity to inhibit substance *P*, bradykinins, histamine, serotonin, and PGs which are mediators in these pains. However, extensive studies are required to explore the analgesic mechanism of the plant extracts.

Acetic acid generates localized inflammatory response for perception of pain. This pain stimulus is responsible for releasing free arachidonic acid from the tissue phospholipids. Acetic acid writhing test is used for the evaluation of peripherally acting analgesics by acid-sensing ion channels, PG pathways, and peritoneal mast cells [2]. Table 3 represents the peripheral analgesic effects of HMPB and PBMPB in acetic acid-induced writhing test. These effects may represent the blocking of peritoneal mast cells, acid-sensing ion channels, and/or the PG pathways.

Among the various acute pain models, tail immersion model is the significant one. Its main function is to assess the activities of central analgesics along with opioid receptor agonists which show extra sensitivity to this model. Supraspinal (*k*_3_, *d*_1_, and *s*_1_) and spinal (*k*_1_, *d*_2_, and *s*_2_) receptors play role in the analgesic activities of opioids. Thermal nociceptive tests such as the tail immersion test get additional sensitivity from opioid *μ* receptor agonists [2]. Table 4 shows the effects of HMPB and PBMPB on latency time in the tail immersion test. Antinociceptive activity of opioids can be initiated in early as well as late phase of pain model [2]. Table 4 point outs that PBMPB showed activity in both early and late phase (i.e., at 30 min and 120 min after the administration of PBMPB) that relates the involvement of opioids. In addition, HMPB 200 mg/kg showed activity in early phase (i.e., at 30 min after the administration of HMPB 200 mg/kg) which contradicts the involvement of opioids. So, broad studies are needed to elucidate the exact pain inhibitory mechanism of actions of the plant extracts.

However, spinal and supraspinal reflexes are also treated with hot plate model. It is also used for the assessment of the mechanism of central analgesics which are opioids in nature [2, 3, 28–30]. Table 5 shows the effects of HMPB and PBMPB on response latency in hot plate test. HMPB and PBMPB 200 mg/kg showed activities in both early and late phase (i.e., at 30 min, 120 min, and 180 min after the administration of HMPB and PBMPB 200 mg/kg) that relate the involvement of opioids. Besides, early and late phase activities of HMPB 400 mg/kg were found at 30 min and 180 min after the administration of HMPB 400 mg/kg which also relate the involvement of opioids. Nevertheless, comprehensive works are essential for exploring the precise mechanism of actions of the pain inhibition by HMPB and PBMPB, respectively.

Prevalence of diarrhea can be controlled traditionally by using* Microcos paniculata*. Intestinal mucosa can experience inflammation as well as irritation through the active component of castor oil named ricinoleic acid. After the irritation of intestinal mucosa, peristaltic movement of the small intestine is prompted. This event leads to alteration in the electrolytic permeability of the intestinal mucosa. After that, secretion and motility of gastrointestinal tract are activated through the release of PGs. As a result, absorption of sodium and potassium ions is reduced, which sequentially lessens the function of Na^+^, K^+^-ATPase in colon plus small intestine [5, 7, 8]. Our results showed that HMPB and PBMPB significantly inhibited (^*∗*^ *P* < 0.05, versus control) castor oil induced diarrhea in mice (Table 6) which may be due to the inhibition of electrolyte permeability of the intestine and prostaglandin release.

Rise of two events such as gathering of fluid in the intestinal lumen and its movement from proximal to the distal intestine occurs after the oral administration of magnesium sulphate. Discharge of nitric oxide and cholecystokinin from duodenal mucosa ensues after its oral administration. Therefore, two consecutive results come about. One is the rise of secretion and motility of small intestine. Another is the inhibition of reabsorption of NaCl and water that occurs from the previous case [7]. HMPB and PBMPB were effective in reducing diarrhea that was expected due to increase in electrolyte and water reabsorption from the gastrointestinal tract.

## 5. Conclusion

From the existing study, it could be suggested that hydromethanol (HMPB) and petroleum benzene extract of* Microcos paniculata* barks (PBMPB) might possess antinociceptive and antidiarrheal activity. Nevertheless, further quantitative chemical studies are now under way to isolate and determine the structure of the active constituents. Similarly, we are seeking out biological testing of the specific compounds thought to be responsible for antinociceptive and antidiarrheal activities presented in the HMPB and PBMPB. Again, genotoxicity study of this plant should be carried out for safety evaluation, though in the present study the plant extracts did not show any acute oral toxicity.

## Figures and Tables

**Table 1:** Phytochemical screening of HMPB and PBMPB.

Phytoconstituents	Test name	Observation of various extracts	
		HMPB	PBMPB
Carbohydrates	Molisch's test	−	−
	Fehling's test	−	−

Alkaloids	Mayer's test	−	−
	Wagner's test	−	−
	Dragendorff's test	−	−

Saponins	Frothing test	+	+

Tannins	FeCl_3_ test	−	−

Flavonoids	Alkali test	−	−
	Pew's test	+	−

Triterpenoids	Salkowski's test	+	+

Glycosides	General test	−	−
	Baljet test	−	−

Anthraquinones	NH_4_OH test	−	−

+: presence of specific phytoconstituents; −: absence of specific phytoconstituents.

**Table 2:** Effect of standard, HMPB, and PBMPB in formalin-induced paw licking test.

Group	Dose	Licking in acute phase (s)	Inhibition (%)	Licking in delayed phase (s)	Inhibition (%)
Control	10 mL/kg	220.40 ± 2.16	0.00 ± 0.00	75.80 ± 1.43	0.00 ± 0.00
DS	100 mg/kg	142.60 ± 3.40^*∗*$*θ*□●^	35.31 ± 1.35^*∗*$*θ*□●^	7.40 ± 0.51^*∗*$*θ*□^	90.27 ± 0.49^*∗*$*θ*□^
HMPB	200 mg/kg	112.40 ± 2.50^*∗*#*θ*□●^	49.02 ± 0.73^*∗*#*θ*□●^	62.40 ± 1.03^*∗*#*θ*□●^	17.60 ± 1.64^*∗*#*θ*□●^
HMPB	400 mg/kg	53.20 ± 2.13^*∗*#$^	75.87 ± 0.90^*∗*#$^	45.20 ± 1.16^*∗*#$□●^	40.35 ± 1.30^*∗*#$□●^
PBMPB	200 mg/kg	58.60 ± 1.50^*∗*#$^	73.40 ± 0.76^*∗*#$^	22.80 ± 1.36^*∗*#$*θ*●^	70.01 ± 1.22^*∗*#$*θ*●^
PBMPB	400 mg/kg	52.40 ± 0.87^*∗*#$^	76.22 ± 0.39^*∗*#$^	10.20 ± 0.37^*∗*$□^	86.56 ± 0.27^*∗*$□^

Values of the first and second 5 min are presented as mean ± standard error. *n* = 5 mice in each group.  ^*∗*^ *P* < 0.05, versus control (Dunnett's *t*-test); ^#^ *P* < 0.05, versus DS 100 mg/kg; ^$^ *P* < 0.05, versus HMPB 200 mg/kg; ^*θ*^ *P* < 0.05, versus HMPB 400 mg/kg; ^□^ *P* < 0.05, versus PBMPB 200 mg/kg; ^●^ *P* < 0.05, versus PBMPB 400 mg/kg (pairwise comparison by post hoc Tukey's HSD test).

**Table 3:** Effect of standard, HMPB, and PBMPB in acetic acid induced writhing test.

Group	Dose	Number of writhing processes	Inhibition (%)
Control	10 mL/kg	36.20 ± 1.50	0.00 ± 0.00
DS	100 mg/kg	4.40 ± 0.40^*∗*$*θ*□●^	87.68 ± 1.36^*∗*$*θ*□●^
HMPB	200 mg/kg	17.00 ± 0.32^*∗*#*θ*□●^	52.75 ± 1.95^*∗*#*θ*□●^
HMPB	400 mg/kg	9.40 ± 0.51^*∗*#$□●^	73.98 ± 1.24^*∗*#$□●^
PBMPB	200 mg/kg	20.20 ± 0.37^*∗*#$*θ*●^	43.94 ± 1.72^*∗*#$*θ*●^
PBMPB	400 mg/kg	13.20 ± 0.37^*∗*#$*θ*□^	63.13 ± 2.51^*∗*#$*θ*□^

Values of the number of writhing processes are presented as mean ± standard error. *n* = 5 mice in each group.  ^*∗*^ *P* < 0.05, versus control (Dunnett's *t*-test); ^#^ *P* < 0.05, versus DS 100 mg/kg; ^$^ *P* < 0.05, versus HMPB 200 mg/kg; ^*θ*^ *P* < 0.05, versus HMPB 400 mg/kg; ^□^ *P* < 0.05, versus PBMPB 200 mg/kg; ^●^ *P* < 0.05, versus PBMPB 400 mg/kg (pairwise comparison by post hoc Tukey's HSD test).

**Table 4:** Effect of standard, HMPB, and PBMPB in tail immersion test.

Group	Dose	Latency time (s)				
		0 min	+30 min	+60 min	+120 min	+180 min
Control	10 mL/kg	1.40 ± 0.24	2.60 ± 0.24	2.20 ± 0.20	1.60 ± 0.24	1.40 ± 0.24
Tramadol	10 mg/kg	2.20 ± 0.37^□●^	4.40 ± 0.24^*∗*$*θ*□●^	5.80 ± 0.20^*∗*$*θ*□●^	4.40 ± 0.24^*∗*$*θ*□^	2.60 ± 0.24^*∗*$*θ*●^
HMPB	200 mg/kg	1.80 ± 0.20^□●^	2.80 ± 0.20^#□●^	1.80 ± 0.20^#●^	1.60 ± 0.24^#□●^	1.20 ± 0.20^#□●^
HMPB	400 mg/kg	2.80 ± 0.20^*∗*□●^	2.20 ± 0.20^#□●^	2.40 ± 0.24^#●^	1.80 ± 0.20^#□●^	1.60 ± 0.24^#□●^
PBMPB	200 mg/kg	4.80 ± 0.20^*∗*#$*θ*^	5.40 ± 0.24^*∗*#$*θ*●^	2.40 ± 0.24^#●^	3.20 ± 0.20^*∗*#$*θ*●^	2.60 ± 0.24^*∗*$*θ*●^
PBMPB	400 mg/kg	5.40 ± 0.24^*∗*#$*θ*^	6.60 ± 0.31^*∗*#$*θ*□^	3.20 ± 0.20^*∗*#$*θ*□^	4.20 ± 0.20^*∗*$*θ*□^	3.60 ± .024^*∗*#$*θ*□^

Latency time values are presented as mean ± standard error. *n* = 5 mice in each group. 0 min means 30 min before drug administration and +30 min, +60 min, +120 min, and +180 min indicate 30, 60, 120, and 180 min after drug administration, respectively.  ^*∗*^ *P* < 0.05, versus control (Dunnett's *t*-test); ^#^ *P* < 0.05, versus tramadol 10 mg/kg; ^$^ *P* < 0.05, versus HMPB 200 mg/kg; ^*θ*^ *P* < 0.05, versus HMPB 400 mg/kg; ^□^ *P* < 0.05, versus PBMPB 200 mg/kg; ^●^ *P* < 0.05, versus PBMPB 400 mg/kg (pairwise comparison by post hoc Tukey's HSD test).

Tests of within-subjects effects conducted by repeated measure analysis of variance reveal that for the factor “time” calculated *F* = 45.00 for all methods and *P* value = 0.000 in every case. So time is highly significant at any level of significance.

**Table 5:** Effect of standard, HMPB, and PBMPB in hot plate test.

Group	Dose	Response latency period (s)				
		0 min	+30 min	+60 min	+120 min	+180 min
Control	10 mL/kg	2.23 ± 0.02	2.24 ± 0.01	2.19 ± 0.01	2.21 ± 0.01	2.25 ± 0.00
Tramadol	10 mg/kg	2.34 ± 0.01^*∗*$*θ*□●^	5.25 ± 0.01^*∗*$*θ*□●^	6.65 ± 0.02^*∗*$*θ*□●^	6.46 ± 0.01^*∗*$*θ*□●^	3.53 ± 0.13^*∗*$*θ*□●^
HMPB	200 mg/kg	8.15 ± 0.01^*∗*#*θ*□●^	8.73 ± 0.01^*∗*#*θ*□●^	5.84 ± 0.01^*∗*#*θ*□●^	6.39 ± 0.01^*∗*#*θ*□●^	6.65 ± 0.01^*∗*#*θ*□●^
HMPB	400 mg/kg	6.18 ± 0.01^*∗*#$□●^	8.08 ± 0.01^*∗*#$□●^	7.35 ± 0.04^*∗*#$□●^	7.21 ± 0.00^*∗*#$□●^	8.75 ± 0.01^*∗*#$□●^
PBMPB	200 mg/kg	7.09 ± 0.03^*∗*#$*θ*●^	7.72 ± 0.05^*∗*#$*θ*●^	3.32 ± 0.01^*∗*#$*θ*●^	3.86 ± 0.01^*∗*#$*θ*●^	4.54 ± 0.01^*∗*#$*θ*●^
PBMPB	400 mg/kg	9.46 ± 0.02^*∗*#$*θ*□^	7.20 ± 0.03^*∗*#$*θ*□^	4.66 ± 0.01^*∗*#$*θ*□^	5.34 ± 0.01^*∗*#$*θ*□^	2.98 ± 0.00^*∗*#$*θ*□^

Response latency values are presented as mean ± standard error. *n* = 5 mice in each group. 0 min means 30 min before drug administration; +30 min, +60 min, +120 min, and +180 min indicate 30, 60, 120, and 180 min after drug administration, respectively.  ^*∗*^ *P* < 0.05, versus control (Dunnett's *t*-test); ^#^ *P* < 0.05, versus tramadol 10 mg/kg; ^$^ *P* < 0.05, versus HMPB 200 mg/kg; ^*θ*^ *P* < 0.05, versus HMPB 400 mg/kg; ^□^ *P* < 0.05, versus PBMPB 200 mg/kg; ^●^ *P* < 0.05, versus PBMPB 400 mg/kg (pairwise comparison by post hoc Tukey's HSD test).

Tests of within-subjects effects conducted by repeated measure analysis of variance reveal that for the factor “time” calculated *F* = 3506.30 for all methods and *P* value = 0.000 in every case. So time is highly significant at any level of significance.

**Table 6:** Effect of standard, HMPB, and PBMPB in castor oil-induced diarrheal test.

Group	Dose	Number of diarrheal feces	% of inhibition of diarrhea
Control	10 mL/kg	9.80 ± 0.37	0.00 ± 0.00
Loperamide HCl	3 mg/kg	1.40 ± 0.24^*∗*$*θ*□●^	85.74 ± 2.37^*∗*$*θ*□●^
HMPB	200 mg/kg	5.80 ± 0.37^*∗*#$●^	40.65 ± 3.82^*∗*#$●^
HMPB	400 mg/kg	3.60 ± 0.24^*∗*#*θ*□●^	62.95 ± 3.23^*∗*#*θ*□●^
PBMPB	200 mg/kg	8.40 ± 0.24^*∗*#$*θ*□^	13.68 ± 4.65^*∗*#$*θ*□^
PBMPB	400 mg/kg	6.00 ± 0.32^*∗*#$●^	38.24 ± 4.39^*∗*#$●^

Values are presented as mean ± standard error. *n* = 5 mice in each group.  ^*∗*^ *P* < 0.05, versus control (Dunnett's *t*-test); ^#^ *P* < 0.05, versus Loperamide HCl 3 mg/kg; ^$^ *P* < 0.05, versus HMPB 200 mg/kg; ^*θ*^ *P* < 0.05, versus HMPB 400 mg/kg; ^□^ *P* < 0.05, versus PBMPB 200 mg/kg; ^●^ *P* < 0.05, versus PBMPB 400 mg/kg (pairwise comparison by post hoc Tukey's HSD test).

**Table 7:** Effect of standard, HMPB, and PBMPB in MgSO_4_ induced diarrheal test.

Group	Dose	Number of diarrheal feces	% of inhibition of diarrhea
Control	10 mL/kg	7.80 ± 0.37	0.00 ± 0.00
Loperamide HCl	3 mg/kg	1.80 ± 0.20^*∗*$□^	76.98 ± 2.40^*∗*$□^
HMPB	200 mg/kg	3.60 ± 0.24^*∗*#^	52.98 ± 4.96^*∗*#^
HMPB	400 mg/kg	2.80 ± 0.20^*∗*^	63.69 ± 3.35^*∗*^
PBMPB	200 mg/kg	3.60 ± 0.24^*∗*#^	53.61 ± 3.35^*∗*#^
PBMPB	400 mg/kg	2.40 ± 0.40^*∗*^	68.13 ± 6.13^*∗*^

Values are presented as mean ± standard error. *n* = 5 mice in each group.  ^*∗*^ *P* < 0.05, versus control (Dunnett's *t*-test); ^#^ *P* < 0.05, versus Loperamide HCl 3 mg/kg; ^$^ *P* < 0.05, versus HMPB 200 mg/kg; ^□^ *P* < 0.05, versus PBMPB 200 mg/kg (pairwise comparison by post hoc Tukey's HSD test).
